# A signal-diffusion-based unsupervised contrastive representation learning for spatial transcriptomics analysis

**DOI:** 10.1093/bioinformatics/btae663

**Published:** 2024-11-15

**Authors:** Nan Chen, Xiao Yu, Weimin Li, Fangfang Liu, Yin Luo, Zhongkun Zuo

**Affiliations:** School of Computer Engineering and Science, Shanghai University, Shanghai 200444, China; School of Computer Engineering and Science, Shanghai University, Shanghai 200444, China; School of Computer Engineering and Science, Shanghai University, Shanghai 200444, China; School of Computer Engineering and Science, Shanghai University, Shanghai 200444, China; School of Life Sciences, East China Normal University, Shanghai 200241, China; Department of General Surgery, The Second Xiangya Hospital, Changsha 410011, China

## Abstract

**Motivation:**

Spatial transcriptomics allows for the measurement of high-throughput gene expression data while preserving the spatial structure of tissues and histological images. Integrating gene expression, spatial information, and image data to learn discriminative low-dimensional representations is critical for dissecting tissue heterogeneity and analyzing biological functions. However, most existing methods have limitations in effectively utilizing spatial information and high-resolution histological images. We propose a signal-diffusion-based unsupervised contrast learning method (SDUCL) for learning low-dimensional latent embeddings of cells/spots.

**Results:**

SDUCL integrates image features, spatial relationships, and gene expression information. We designed a signal diffusion microenvironment discovery algorithm, which effectively captures and integrates interaction information within the cellular microenvironment by simulating the biological signal diffusion process. By maximizing the mutual information between the local representation and the microenvironment representation of cells/spots, SDUCL learns more discriminative representations. SDUCL was employed to analyze spatial transcriptomics datasets from multiple species, encompassing both normal and tumor tissues. SDUCL performed well in downstream tasks such as clustering, visualization, trajectory inference, and differential gene analysis, thereby enhancing our understanding of tissue structure and tumor microenvironments.

**Availability and implementation:**

https://github.com/WeiMin-Li-visual/SDUCL.

## 1 Introduction

The spatial distribution of different cells within complex tissues is closely related to their biological functions (Moses and Pachter 2022). Although single-cell RNA sequencing (scRNA-seq) technology has enabled high-throughput gene expression profiling, it disrupts tissue integrity during sample preparation, preventing the retention of precise cellular arrangements and spatial adjacencies within the original tissue. However, preserving this spatial information can provide multidimensional structural insights, aiding in a deeper understanding of how gene expression in cells is influenced by their surrounding environment. Spatial transcriptomics, a next-generation technology based on sequencing and imaging methods, addresses this limitation. It captures cellular heterogeneity while preserving spatial location information (Rao *et al.* 2021). Therefore, spatial transcriptomics holds significant value in revealing tissue structure and function and understanding the mechanisms of development and disease.

The high dimensionality and complexity of spatial transcriptomic data, and the spatial information it contains significantly increase the challenge of data analysis. Integrating gene expression, spatial information, and image data to learn informative and discriminative low-dimensional representations for each cell or spot helps to uncover potential patterns and structures within the data, providing a more reliable foundation for subsequent downstream tasks. However, early analysis methods, such as Seurat (Satija *et al.* 2015) is designed for scRNA-seq analysis, primarily focusing on gene expression data and ignored the spatial arrangement of cells. Although Spacell (Tan *et al.* 2020) combines gene expression and image information, it still fails to fully leverage the specific spatial locations of cells within the tissue.

In recent years, numerous algorithms synthesizing spatial information have been developed to better understand the relationship between cellular spatial distribution and function. For example, Giotto (Dries *et al.* 2021) and SC-MEB (Yang *et al.* 2022) model gene expression based on Markov models, while BayesSpace (Zhao *et al.* 2021) employs a fully Bayesian statistical approach, treating spatial information as prior knowledge. However, these statistical model-based methods are limited in performance as they fail to capture the nonlinear characteristics of gene expression. To overcome these limitations, a series of deep learning methods have been proposed, such as the Graph Neural Network (GNN)-based methods CCST (Li *et al.* 2022), STAGATE (Dong and Zhang 2022), and SEDR (Xu *et al.* 2024). Research has demonstrated that histopathological images can predict gene expression (Zeng *et al.* 2022). Hence, there are methods such as stLearn (Pham 2020) and SpaGCN (Hu *et al.* 2021) that integrate neighborhood information and morphological features. Besides, DeepST (Xu *et al.* 2022) further integrates image features, gene expression, and spatial location. Despite these methods effectively use spatial information, histological images, and gene expression, they often fall short in fully exploiting the structural information of unlabeled data, particularly the topological features of cell or spot spatial relationships. To address the limited labeling in spatial transcriptomics data, some methods employed contrastive learning to leverage the data’s inherent structure and attributes for learning low-dimensional representations. For instance, conST (Zong 2022) integrates multimodal data and performs contrastive learning at the point, sub-cluster, and global levels. ConGI (Zeng *et al.* 2023) introduces three contrastive loss functions encompassing gene expression, images, and image-to-gene expression to learn joint representations of the two modalities. GraphST (Long *et al.* 2023) leverages spatial information and gene expression through graph self-supervised contrastive learning. In spite of that contrastive learning methods can extract deep features from unlabeled data, most existing approaches have not fully considered cell interactions within their local microenvironment.

In this study, we propose a novel method called signal-diffusion-based unsupervised contrast learning method (SDUCL), a signal-diffusion-based unsupervised contrastive learning method. SDUCL first utilizes a pre-trained deep neural network model to extract morphological features from histological images. Subsequently, a spatial topology feature extractor is designed to thoroughly capture both local and global characteristics of the spatial relationships between cells or spots. By integrating image features, spatial topological features, and gene expression features, we obtain an augmented feature representation. Traditional contrastive learning methods often aggregate directly connected nodes or all nodes within a graph into a single global representation, neglecting the complexity of biological network microenvironments. The complexity and diversity of these microenvironments, as well as the unclear mechanisms of biological signal diffusion, present significant challenges in discovering microenvironments. To address these issues, we propose a signal diffusion microenvironment discovery algorithm. This algorithm identifies the microenvironments of nodes within a spatial relational graph, capturing intricate interaction information among cells. SDUCL then utilizes a graph convolutional network (GCN) to learn both local and microenvironment representations, maximizing the mutual information between them to develop more discriminative low-dimensional representations. To validate the efficacy of SDUCL, we performed experiments on normal and tumor datasets from several species including human, mouse, and phalaenopsis. The results demonstrate that the representation learned by SDUCL effectively support various downstream tasks, such as clustering, visualization, trajectory inference, and differential gene analysis.

## 2 Materials and methods

### 2.1 Datasets description

To access the performance of SDUCL, we applied SDUCL to seven spatial transcriptome datasets sourced from multiple species and different sequencing platforms, specifically including: (1) dorsolateral prefrontal cortex (DLPFC) dataset (Maynard *et al.* 2021): from human DLPFC, comprising 12 brain slices from three subjects; (2) mouse brain dataset: collected from sagittal slices of mouse brain tissue; (3) breast cancer (BRCA) dataset: ductal carcinoma *in situ*, lobular carcinoma *in situ*, invasive carcinoma, covering four major morphological regions with 20 subregions; (4) pancreatic ductal adenocarcinoma (PDAC) dataset (Moncada *et al.* 2020): pancreatic ductal adenocarcinoma, containing four different regions; (5) Phalaenopsis dataset (Liu *et al*. 2022): three samples of orchid inflorescence tissue in sagittal sections, covering early developmental stages from inflorescence meristem formation to nearing maturity of floral organs. (6) Mouse olfactory bulb dataset: This dataset originates from mouse olfactory bulb tissue and was generated using Stereo-seq technology (Chen *et al*. 2022). The DAPI-stained image has been annotated into seven distinct layers. (7) Mouse hypothalamic preoptic dataset (Allen *et al.* 2023): This dataset is derived from the mouse hypothalamic preoptic region and was obtained using the MERFISH technique (Chen *et al.* 2015), an imaging-based spatially resolved transcriptomics technology (see Supplementary data for detailed information and processing).

### 2.2 The architecture of SDUCL

**Figure 1.**
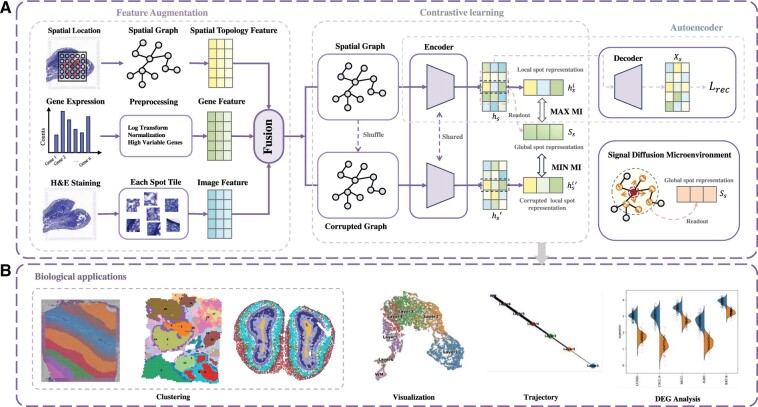
Overview of the SDUCL. (A) The input consists of gene expression data, spatial location, and histological images. A spatial graph is constructed to extract topological feature, and a pretrained network extracts image feature. These gene, spatial, and image features are fused to obtain augmented features. A signal-diffusion-based unsupervised contrastive learning method is then used to learn low-dimensional representations for each cell/spot. (B) Biological applications include spatial clustering, visualization, trajectory analysis, differential expression gene (DEG) analysis, and other related analyses.

SDUCL is a signal-diffusion-based unsupervised contrastive learning method, which enhances features by integrating histological morphological features, spatial information, and gene expression to model the low-dimensional representation of the spatial transcriptome. As shown in Fig. 1A, the process begins with feature augmentation of the raw data, including extracting image features, spatial topological features, and gene expression features, and obtaining a comprehensive data representation through tensor fusion module. Next, the augmented features are utilized to learn low-dimensional representations through unsupervised contrast learning.

To generate training samples for contrastive learning, we randomly shuffle the original spatial relationship graph to create a corrupted graph. To fully capture the information within the complex microenvironments of biological networks, we propose a signal diffusion microenvironment discovery algorithm based on network topology characteristics. This algorithm identifies the microenvironments of nodes in the spatial relationship graph. Through GCNs, we aggregate local and global microenvironment information to better preserve both local neighborhoods and global context. The local and global representations of nodes in the original graph constitute positive sample pairs, while the local representation in the corrupted graph and the global representation of the original graph constitute negative sample pairs. By maximizing the mutual information between positive sample pairs while minimizing the mutual information between negative sample pairs, we learn node representations that incorporate both node features and contextual information from their microenvironments.

Finally, the learned low-dimensional representations are applied to various downstream analysis tasks, such as clustering, data visualization, trajectory inference, and differential gene analysis (see Fig. 1B).

#### 2.2.1 Spatial relationship extractor

Spatial coordinate information is a key feature in spatial transcriptomics data. In order to make full use of the spatial coordinate information, we constructed a spatial relation graph $G=\left(V,E\right)$ to represent the spatial proximity between different spots, where V represents the set of points and E represents the set of connected edges. Specifically, we first calculated the Euclidean distance between different spots based on the spatial coordinate, and employed the K-nearest neighbor algorithm to find the K-nearest neighbors for each spot. We define the two-dimensional adjacency matrix of spatial relationship graph as $A=(a_{ij}{)}_{N_{\mathrm{spot}}\times N_{\mathrm{spot}}}$, when $a_{ij}=a_{ji}=1$ indicates an edge between spots i and j, otherwise $a_{ij}=a_{ji}=0$, and $N_{\mathrm{spot}}$ is the number of spots. In this study, the default value of K is set to 6.

#### 2.2.2 Feature augmentation module

##### 2.2.2.1 Image feature extractor

To fully utilize the histopathological image features, we employ an image feature extractor, $F_{\mathrm{ResNet}50}$, to capture the morphological features of each spot from image patches. The backbone network of the image feature extractor is ResNet50 (He 2016). Considering the limited amount of images available for training, we retain the pretrained weights of ResNet50. The input to the image feature extractor consists of image patches obtained by cropping the entire histopathological image, with each patch corresponding to a respective gene expression matrix. This ensures that the fused features are not only biologically relevant but also reflective of the tissue’s morphological characteristics. If we represent the entire histopathological image as I, then through a cropping operation C, we obtain a series of image patches$\;\{x_{1},x_{2},\ldots ,x_{N_{\mathrm{spot}}}\}$, where each $x_{i}$ represents an image patch, and $N_{\mathrm{spot}}\;$is the total number of image patches, corresponding to the total number of spots. These cropped images are then fed into the image feature extractor to obtain the image features$\;E_{i}$:

(1) $$C(I)=\{x_{1},x_{2},\ldots ,x_{n}\}.$$

(2) $$E_{i}=F_{\mathrm{ResNet}50}\left(C\left(I\right)\right).$$

##### 2.2.2.2 Spatial topology feature extractor

To gain a deeper understanding of the complex spatial relationships within tissues, we design a spatial topology feature extractor to uncover the interaction strength between nodes in the spatial relationship graph, enabling a more thorough extraction of both local and global features from the graph (Yu *et al.* 2024a). This spatial feature extractor is based on the concept of Katz index (Martínez *et al.* 2017), which measures the influence and importance of a node in the network topology by analyzing the number and length of paths between nodes. The definition is given in Equation (3), where A is the adjacency matrix of the spatial relational graph, $A^{l}\;$represents all paths of length l, and β is the attenuation factor. The input of the spatial feature extractor is the adjacency matrix of the spatial relational graph. By calculating the spatial topological properties, we quantify the degree of interactions among nodes in the spatial relational graph. The spatial topology feature extractor considers all paths between node pairs, with shorter paths indicating stronger relationships, while the influence diminishes as path length increases. Therefore, we can capture not only the direct connections between each spot and its immediate neighbors but also quantify the indirect interactions between distant spots connected via multi-step paths.

(3) $$E_{t}=\sum_{l=1}^{\infty }\;\beta^{l}\cdot (A^{l}{)}_{ij}.$$

##### 2.2.2.3 Tensor fusion module

In the tensor fusion module, we use the multidimensional property of tensor to fuse the gene expression features, image features, and spatial topology features of spatial transcriptome data into a more comprehensive feature tensor to obtain a richer representation of information and improve the generalization ability (Yu *et al.* 2024b). As in Equation (4), where $E_{g}$ denotes gene expression features, $E_{i}$ denotes image features, $E_{s}$ denotes spatial topology features, and ⊕ denotes splicing operation, the original information of each feature is preserved by tensor fusion and the correlation between different features is captured.

(4) $$F=E_{g}⊕E_{i}⊕E_{s}.$$

#### 2.2.3 Graph autoencoder

The graph autoencoder module consists of an encoder and a decoder, designed to learn meaningful low-dimensional representations from the input data. The input to the encoder is the spatial relation graph G and the augmented feature tensor F, with the output being the reconstructed feature tensor. More specifically, we employ a GCN as the encoder, which updates the node representation of each spot by iteratively aggregating the features of neighboring nodes. In the encoder, we use a nonlinear activation function σ, a trainable weight matrix W, and a bias vector b to compute the representation at each layer. Layer l in the encoder can be represented by the following equation:

(5) $$h_{s}^{\left(l\right)}=\sigma ({\hat{D}}^{-\frac{1}{2}}\hat{A}{\hat{D}}^{-\frac{1}{2}}h_{s}^{\left(l-1\right)}W_{e}^{\left(l-1\right)}+b_{e}^{\left(l-1\right)}).$$

where $h_{s}^{\left(l\right)}$ denotes the output representation of layer l, $\hat{A}=A+I$ is the adjacency matrix plus the self-connections, $\hat{D}$ is the degree matrix of $\hat{A}$, $h_{s}^{\left(0\right)}=F$ is the input feature tensor, $W_{e}^{\left(l\right)}$ and $b_{e}^{\left(l\right)}$ denote trainable weight matrices and bias vectors, and $\sigma \left(\cdot \right)$ is the nonlinear activation function (ReLU);

The decoder aims to map the latent representations $h_{s}\;$learned by the encoder back to the original feature space to reconstruct the input feature tensor. We adopt a symmetric architecture to invert the encoder’s output, mapping the latent representations back to the space of the original features. The representation l at layer in the decoder can be expressed by the following equation:

(6) $$X_{s}^{\left(l\right)}=\sigma ({\hat{D}}^{-\frac{1}{2}}\hat{A}{\hat{D}}^{-\frac{1}{2}}X_{s}^{\left(l-1\right)}W_{d}^{\left(l-1\right)}+b_{d}^{\left(l-1\right)}).$$

where $X_{s}^{\left(l\right)}$ denotes the reconstructed gene expression feature at layer l, $X_{s}^{\left(0\right)}$ is the output representation $h_{s}$ of the encoder, and $W_{d}^{\left(l\right)}$ and $b_{d}^{\left(l\right)}$ denote trainable weight matrix and bias vectors.

Finally, the model is trained by constructing a self-reconfiguration loss that minimizes the difference between the original input data and the reconstructed data:

(7) $$L_{\text{rec}}=\sum_{i=1}^{N_{\text{spot}}}\;||f_{i}-x_{i}|{|}_{2}^{2}.$$

where $f_{i}$ and $x_{i}$ are the original input feature tensor and the reconstructed feature tensor of point i, respectively.

#### 2.2.4 Signal-diffusion-based unsupervised contrastive learning

To fully account for the interactions of cells within their microenvironments and to make the representation $h_{s}\;$more discriminative, we propose an unsupervised contrastive learning strategy based on signal diffusion. A signal diffusion microenvironment discovery algorithm is designed to identify the complex microenvironments of nodes within a spatial relationship graph, capturing the interaction information of the cellular microenvironment. A global representation for each node is defined by aggregating the representations of the nodes in the microenvironments. This method ensures that the learned representations contain richer and more informative features by maximizing the mutual information between the local and global representations.

##### 2.2.4.1 Data samples construction

we employ the corruption function C to generate a corrupted neighborhood graph for the subsequent contrastive learning process. Specifically, given the original spatial relation graph G and the augmented feature tensor F, we create the corrupted graph by randomly shuffling the feature expression vectors without changing original graph’s topology. The corrupted graph and the shuffled feature tensor are denoted as G′ and F′, respectively [Equation (8)].

(8) (G′,F′)=shuffle(G,F).

##### 2.2.4.2 Signal diffusion microenvironment discovery algorithm

To discover the microenvironments of nodes in the spatial relationship graph, a signal diffusion microenvironment discovery algorithm is proposed on the basis of network topology characteristics. This algorithm determines the importance and affiliation of nodes by simulating the diffusion of biological signals in space. Specifically, the algorithm uses a set of source nodes as initial signal sources, aiming to identify the subnetwork most relevant to the source node set, defined as the microenvironment. Each source node has the same initial signal strength, and the biological signal diffuses to adjacent nodes via connecting edges. Subsequently, a list of nodes is generated and ranked according to the accumulated signal strength. Nodes with more connections typically have higher signal strength, while relatively isolated nodes have lower signal strength, thereby identifying the subnetwork most relevant to the original set [Equation (9)].

(9) m=s* exp⁡(-Lα),

(10) $$\exp(X)=\sum_{k=0}^{\infty }\frac{1}{k!}X^{k},$$

where m represents the result vector, s denotes the initial signal source, and L is the graph Laplacian operator, defined as *L* = *D*-*A*, where *D* is a diagonal matrix containing the degree of each node, and *A* is the adjacency matrix of the input network. α is the signal diffusion rate, with a default value of α = 0.1, controlling the extent of signal spread from the source nodes across the network. exp denotes the matrix exponential, and exp⁡(-Lα) simulates the signal diffusion process within the network.

##### 2.2.4.3 Node(local)—microenvironment(global) mutual information maximization

To better capture the relationships and features between nodes, SDUCL aims to maximize the mutual information between local node representations and global microenvironment representations. First, given the original spatial relationship graph G and the corrupted graph G′ as inputs, the encoder ε aggregates neighbor information to obtain the local context representations $h_{s}\;$ and $h_{s}’$ for the corresponding nodes. Subsequently, the signal diffusion microenvironment discovery algorithm identifies the microenvironment for each node. Drawing inspiration from Deep Graph Infomax (DGI) (Velickovic 2019), we aggregate representations of different nodes within the subnet to form a global neighborhood microenvironmental representation $S_{s}$. Unlike DGI which captures the entire graph as global information, our global representation is obtained by averaging all node features in the neighborhood microenvironment:

(11) $$S_{s}\;=R(H)=\sigma (\frac{1}{N_{\mathrm{sub}}}\sum_{i=1}^{N_{\mathrm{sub}}}\;h_{s}^{i}).$$

For each node i in the spatial graph, let the node representation $h_{s}$ and the microenvironment representation $S_{s}$ be positive sample pairs, and the corresponding representation $h_{s}\prime$ in the corrupted graph and the microenvironment representation $S_{s}$ be negative sample pairs. To maximize the mutual information of nodes and microenvironments, we score the sample pairs by a bilinear binary classifier apparatus.

(12) $$D(h_{s}^{i},S_{s})=\sigma \left({h_{s}^{i}}^{T}WS_{s}\right).$$

The key idea of contrast learning is to maximize the mutual information between positive pairs while minimizing the mutual information between negative pairs. We use the standard binary cross-entropy loss as the objective function:

(13) $$L_{\mathrm{con}}=-\frac{1}{2N}\left(\sum_{i=1}^{N}\;E_{\left(F,A\right)}\left[\log D\left(h_{s}^{i},S_{s}\right)\right]+\sum_{j=1}^{M}\;E_{\left(F\prime ,A\prime \right)}\left[\log\left(1-D\left(h_{s}^{i}\prime ,S_{s}\right)\right)\right]\right),$$

where, $E_{\left(F,A\right)}$ is the encoder that takes the features F and adjacency matrix A as inputs and outputs the local node embeddings $h_{s}^{i}$. Similarly, $E_{\left(F\prime ,A\prime \right)}$ represents the encoder processing corrupted features F′ and adjacency matrix A′, generating $h_{s}^{i}\prime$. D is a bilinear binary classifier used to maximize mutual information on graph representations.

To make the model more robust, we consider damage maps with the same structure, defining a symmetric contrast loss:

(14) $$L_{\mathrm{con}}^{\prime }=-\frac{1}{2N}\left(\sum_{i=1}^{N}\;E_{\left(F^{\prime },A^{\prime }\right)}\left[\log D\left(h_{s}^{i\prime },S_{s}^{\prime }\right)\right]+\sum_{j=1}^{M}\;E_{\left(F,A\right)}\left[\log\left(1-D\left(h_{s}^{i},S_{s}^{\prime }\right)\right)\right]\right).$$

#### 2.2.5 Model training strategy

The model training process is performed by minimizing the self-reconfiguration loss and the comparison loss. The overall training loss of the model is defined as follows:

(15) $$L=\alpha L_{\mathrm{rec}}+\beta {(L}_{\mathrm{con}}+L_{\mathrm{con}}\prime ),$$

where α and β are weighting factors used to balance the effects of reconstruction loss and contrast loss.

#### 2.2.6 Evaluation metrics for clustering

For spatial transcriptomic data with manual annotations, we will evaluate the spatial clustering performance using four commonly used clustering metrics, including the Adjusted Rand Index (ARI), Normalized Mutual Information (NMI), V-measure, and Purity. In cases lacking manual annotations, we employ two internal evaluation metrics, namely the Silhouette Coefficient (SC) and Davies-Bouldin Index (DBI), to assess clustering (see Supplementary data for detailsclustering method).

## 3 Results

### 3.1 Applications of spatial transcriptomics in normal tissues

#### 3.1.1 Human dorsolateral prefrontal cortex

**Figure 2.**
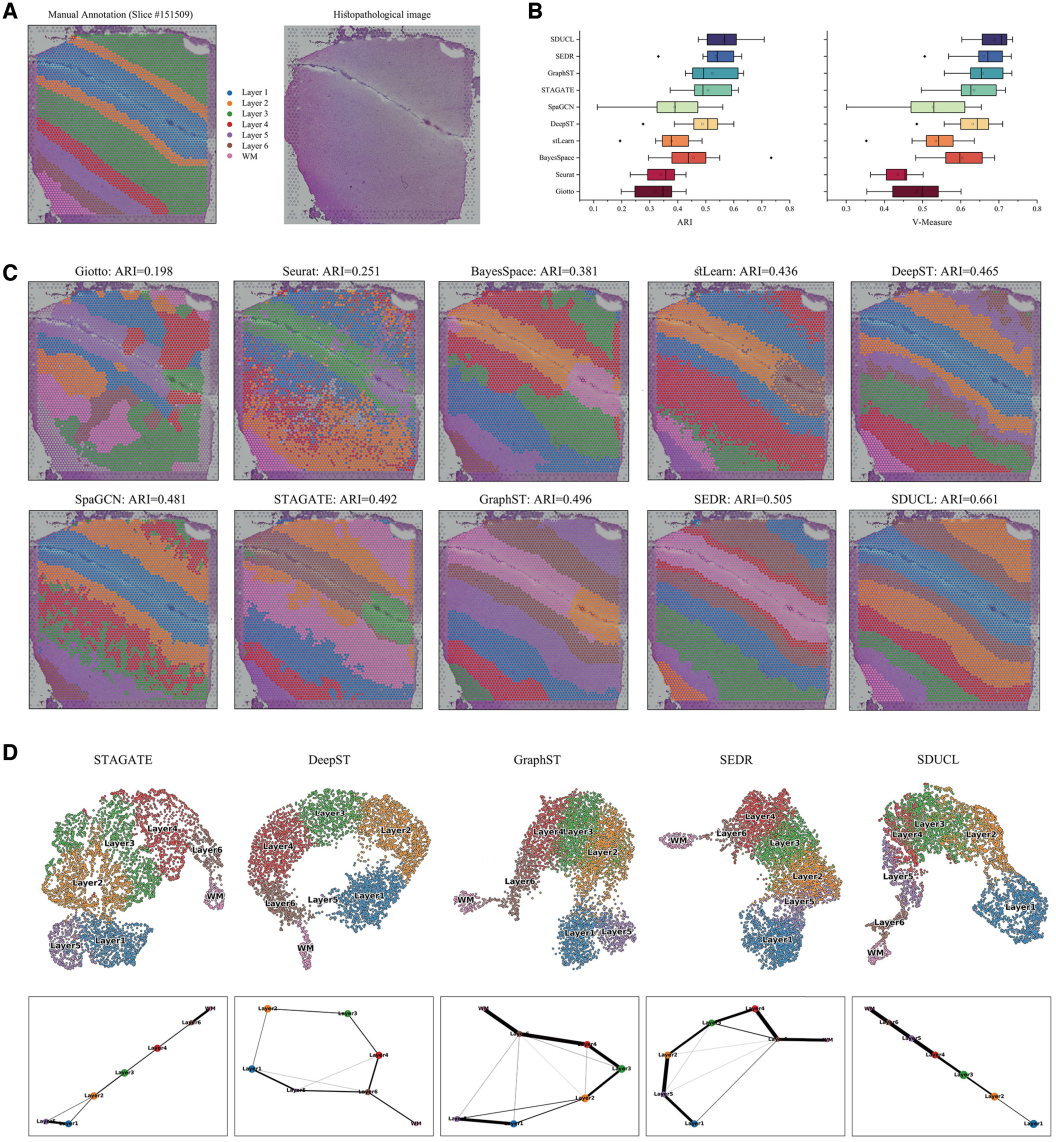
SDUCL improved the identification of layer structures in the DLPFC dataset. (A) Histological images and manually annotation of slice #151509. (B) Boxplots of clustering evaluation metrics for 12 slices from the DLPFC dataset, showcasing the ARI and V-measure for nine baseline methods compared to SDUCL. (C) Clustering results of slice #151509 from the DLPFC dataset. (D) UMAP visualization and PAGA trajectory analysis for STAGATE, DeepST, GraphST, SEDR, and SDUCL. Notably, SpaGCN is not visualized due to its end-to-end methodology.

The quick brown fox jumps over the lazy dog. The quick brown fox jumps over the lazy dog. The quick brown fox jumps over the lazy dog. The quick brown fox jumps over the lazy dog. To quantitatively assess the spatial clustering performance of SDUCL, we first applied it to the DLPFC dataset containing 12 slices, a publicly available 10xVisium spatial transcriptome dataset. Maynard *et al.* (2021) have already manually annotated the cortical layers (L1–L6) and white matter (WM) of the DLPFC dataset based on morphological features and gene expression information (Fig. 2A), which can be served as the basic facts. We compared SDUCL with nine state-of-the-art (SOTA) methods, which contain one non-spatial clustering method (Seurat) and eight spatial clustering methods (Giotto, Seurat, BayesSpace, stLearn, DeepST, SpaGCN, STAGATE, GraphST, SEDR). A comparison of clustering accuracy was conducted across 12 tissue sections using ARI, V-measure, NMI, and purity (Fig. 2B and Supplementary Fig. S3). The results showed that SDUCL outperformed existing SOTA methods, with a median ARI of 0.57, 3% higher than the next best (SEDR) and 8% higher than the third best (GraphST). The NMI, V-measure and Purity exhibited a similar trend (see Supplementary data for clustering method).

To illustrate with slice #151509 (Fig. 2C), which contains 4789 spots and 17 519 genes. We found that SDUCL clearly delineated the boundaries between different cortical layers and showed the best performance on the ARI metric (ARI = 0.661), followed by SEDR (ARI = 0.505). Among the baseline methods, Seurat did not consider spatial information when processing spatial transcriptome data, leading to highly confusing clustering results. The statistical model-based spatial methods (Giotto, BayesSpace, and stLearn) failed to accurately capture fundamental truths. Methods utilizing GCNs, such as DeepST, SpaGCN, STAGATE, GraphST, and SEDR demonstrated superior performance, indicating the effectiveness of GCNs in integrating spatial information. Although stLearn, DeepST, and SpaGCN incorporate histological image information, their performance was not outstanding. This suggested that histological image is complementary to some degree, but the impact might be limited.

To evaluate the effectiveness of the low-dimensional representations learned by SDUCL, we visualized the low-dimensional embeddings of slice #151509 using UMAP and performed trajectory inference with the PAGA algorithm. We compared SDUCL with DeepST, STAGATE, GraphST, and SEDR, which demonstrated good spatial clustering performance. As shown in Fig. 2D, in both UMAP and PAGA plots, all five different methods are better at distinguishing most of the spots from different cortical layers and WM regions. Consistent with the basic facts, SDUCL shows an almost linear developmental trajectory from layer 1 to layer 6 and then to WM. In contrast, the PAGA maps of the other methods were mixed to varying degrees. Consequently, SDUCL can learn effective representations for downstream tasks.

#### 3.1.2 Mouse brain

**Figure 3.**
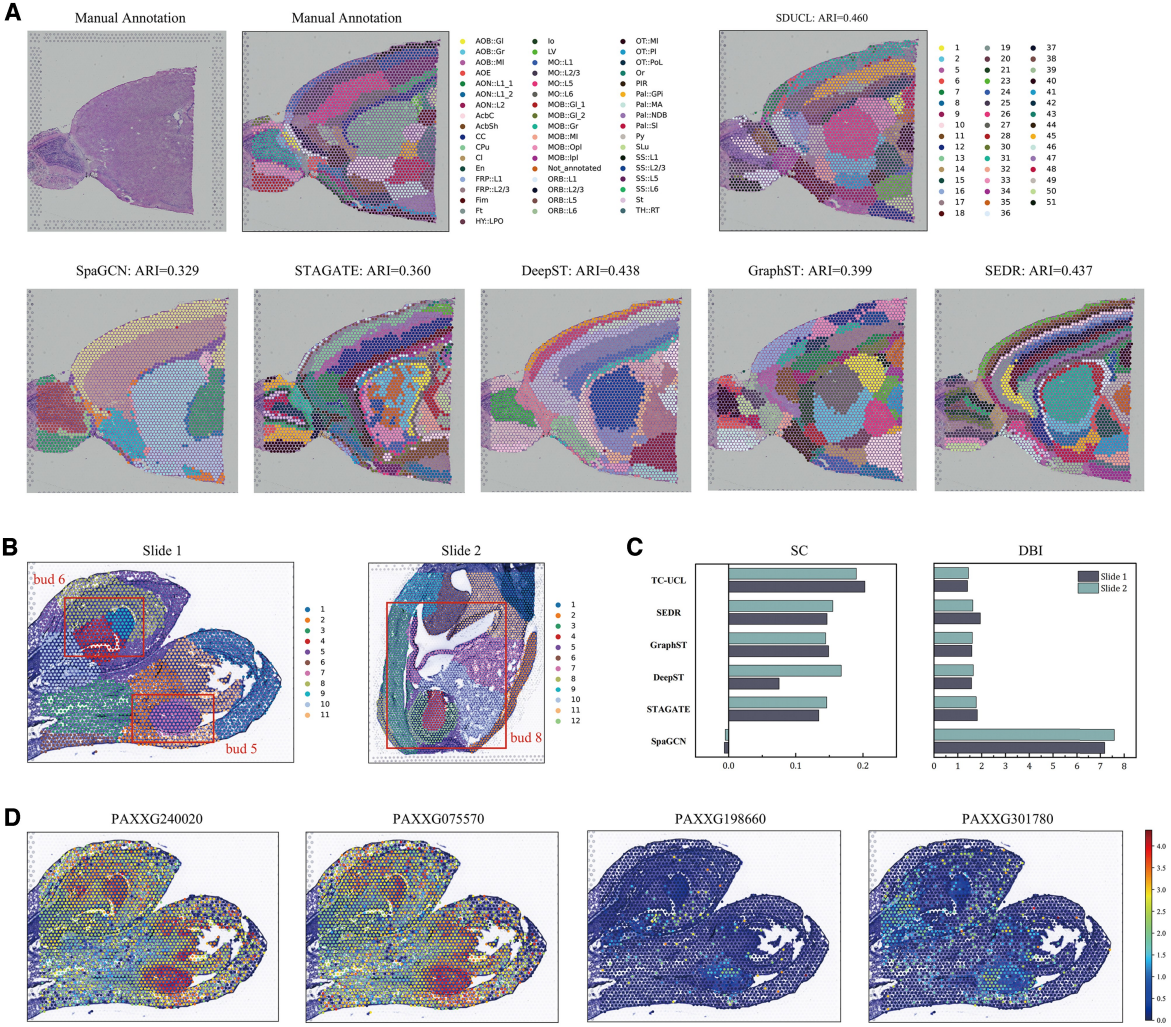
Application of SDUCL to mouse brain and phalaenopsis datasets (A) Histological images and manual annotations of the mouse brain tissue, along with clustering results of DeepST, SpaGCN, STAGATE, GraphST, SEDR, and SDUCL. (B) Clustering results of SDUCL for slices 1 and 2 of the phalaenopsis dataset. (C) Bar graphs of clustering evaluation metrics (SC, DBI) for the phalaenopsis dataset, including five baseline methods and SDUCL. (D) DEGs in cluster 7 of bud 5.

To further explore the versatility of SDUCL, we tested it on complex mouse brain tissue data. The mouse brain sagittal-anterior serial section has 3798 spots and 36 601 genes, which can be divided into 52 subregions (Fig. 3A). We benchmarked SDUCL against SpaGCN, STAGATE, DeepST, GraphST, and SEDR, which showed superior performance as mentioned above. SDUCL achieved the best clustering performance with an ARI of 0.460, representing a 6.3% improvement over the next best algorithm, SEDR. Furthermore, two methods utilizing histological images (SpaGCN and DeepST) fail to delineate the subregions finely, incorrectly grouping them into a single domain. This may be due to the lack of clear spatial domain boundaries in the mouse brain histopathological image. These results indicated that even without clear subregion boundaries in histopathological images, SDUCL can extract discriminative low-dimensional representations, demonstrating superior spatial clustering performance.

#### 3.1.3 Phalaenopsis

Orchidaceae is one of the most species-rich families of angiosperms, comprising approximately 880 genera and over 28 000 species, accounting for about 10% of all angiosperm species (Cai *et al*. 2015). Species in this family show significant diversity in both ecological niches and floral morphology. To explore the generalizability of SDUCL on plant datasets, we applied it to samples of Phalaenopsis covering inflorescence meristematic tissue and nearly mature floral organs. Spatial clustering was first performed on two samples of near-inflorescence meristematic tissue and near-mature floral organs using SpaGCN, STAGATE, DeepST, GraphST, SEDR, and SDUCL (Fig. 3B). Given the lack of manual annotation in the Phalaenopsis dataset, SC and DBI were used as internal metrics for evaluation. As can be seen in Fig. 3C, the clustering effect of the SDUCL algorithm is optimal on both slice 1 and slice 2. The SC reaches 0.203 on Slice 1, which is a 5.6% improvement over the suboptimal SEDR. On Slice 2, the SC reaches 0.19, which is 3.5% higher than the next best DeepST. The lower value of DBI indicates the better clustering effect, and the SDUCL reaches the optimization on both Slice 1 and Slice 2 (Supplementary Figs S4 and S5).

To investigate gene expression in the floral organs during development, differential gene analysis was performed on cluster 7 in slice 1 (Fig. 3D), and 664 differentially expressed genes were detected (|log fold change| ≥ 2; *P*-value < 0.05). These genes were mostly functionally related to growth hormone and cytokinin signaling pathways, and showed different expression patterns in different tissues. Among them, PAXXG240020 was widely expressed across various organs, with the highest expression at the stigma apex and labellum. This high expression might be due to the initiation of anther development typically occurring at the stigma apex. Additionally, PAXXG198660 and PAXG301780 belong to the G-like genes (AGL6-like) within the MADS-box gene family. These G-like genes are crucial for regulating floral meristem characteristics and play a significant role in ovule development (Hsu *et al.* 2021). It can be observed that some of the genes highly expressed in bud 5 showed an overall decrease in expression from bud 5 to bud 8. For instance, in bud8, the expression of PAXXG240020 and PAXXG198660 dropped to undetectable levels, and the expression of PAXG301780 gradually reduced. This demonstrates that dynamic gene expression changes during the development of floral organs.

#### 3.1.4 High-resolution spatial transcriptomics datasets

With the ongoing progress in spatial transcriptomics technologies, both the number of detectable cells and the spatial resolution with tissues are poised to improve substantially. Advanced technologies like Stereo-seq and MERFISH offer resolutions at submicron and even subcellular levels, resulting in the production of large-scale, high-resolution datasets. To assess the performance of SDUCL in handling these high-resolution spatial transcriptomics datasets, we utilized Stereo-seq dataset from mouse olfactory bulb coronal sections and MERFISH dataset from the mouse hypothalamic preoptic region.

**Figure 4.**
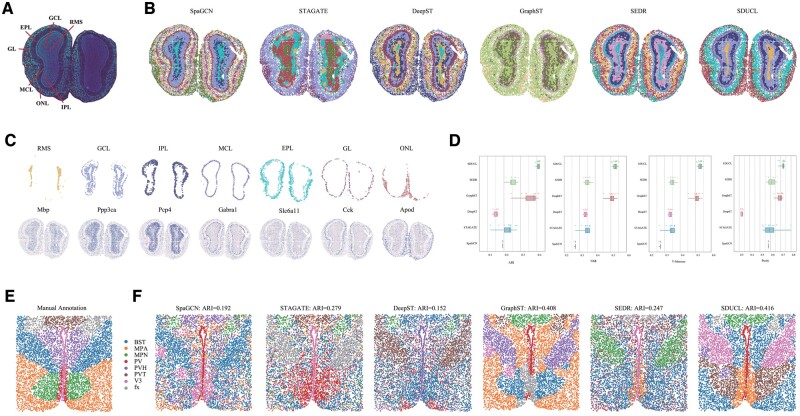
Application of SDUCL on high-resolution datasets. (A) DAPI-stained image of the mouse olfactory bulb dataset, highlighting the layered structure of the olfactory bulb. (B) Clusters identified by six methods (SpaGCN, STAGATE, DeepST, GraphST, SEDR, and SDUCL) on the mouse olfactory bulb Stereo-seq dataset (number of clusters = 10). (C) Visualization of clusters identified by SDUCL along with their corresponding marker gene expressions. (D) Boxplots of evaluation metrics for various methods applied to the mouse hypothalamic preoptic dataset. (E) Manual annotation results of the mouse hypothalamic preoptic dataset. (F) Visualization of clustering results from SpaGCN, STAGATE, DeepST, GraphST, SEDR, and SDUCL.

The coronal mouse olfactory bulb tissue consists of seven layers (Fig. 4A): the olfactory nerve layer (ONL), glomerular layer (GL), external plexiform layer (EPL), mitral cell layer, internal plexiform layer (IPL), granule cell layer (GCL), and the rostral migratory stream (RMS). We applied six methods (SpaGCN, STAGATE, DeepST, GraphST, SEDR, and SDUCL) to partition the olfactory bulb tissue. For consistency, we set the number of clusters to 7, 8, and 10 across all methods (Fig. 4B and Supplementary Fig. S6). The results demonstrated that SpaGCN, DeepST, SEDR, and SDUCL captured regional boundaries more accurately, aligning closer with annotated staining data, while the other methods exhibited some confusion between regions. Notably, SpaGCN, SEDR, and SDUCL more clearly separated the internal structures of the olfactory bulb (RMS, GCL, and IPL), with SDUCL achieving the clearest delineation of the outer layers (ONL, GL, and EPL), showing minimal overlap between clusters. Moreover, SDUCL’s results were validated by known marker genes (Mbp, Ppp3ca, Pcp4, Gabra1, Slc6a11, Cck, and Apod) (Fig. 4C) (Renelt *et al*. 2014).

The preoptic region of the mouse hypothalamus exhibits a complex organizational structure, with diverse regional shapes and adjacency patterns (Fig. 4E). We employed SpaGCN, STAGATE, DeepST, GraphST, and SEDR as benchmark methods for spatial clustering. The results indicated that GraphST and SDUCL achieved superior clustering performance, with ARI values exceeding 0.4, while other algorithms failed to correctly partition the regions (Fig. 4F). For each algorithm, we conducted 10 repeated experiments. Box plots show that the SDUCL algorithm demonstrated greater stability (Fig. 4D).

In summary, the results of these analyses validate the efficacy of SDUCL when applied to high-resolution spatial transcriptomics datasets.

### 3.2 Applications of spatial transcriptomics in tumor tissues

#### 3.2.1 Human pancreatic ductal adenocarcinoma

**Figure 5.**
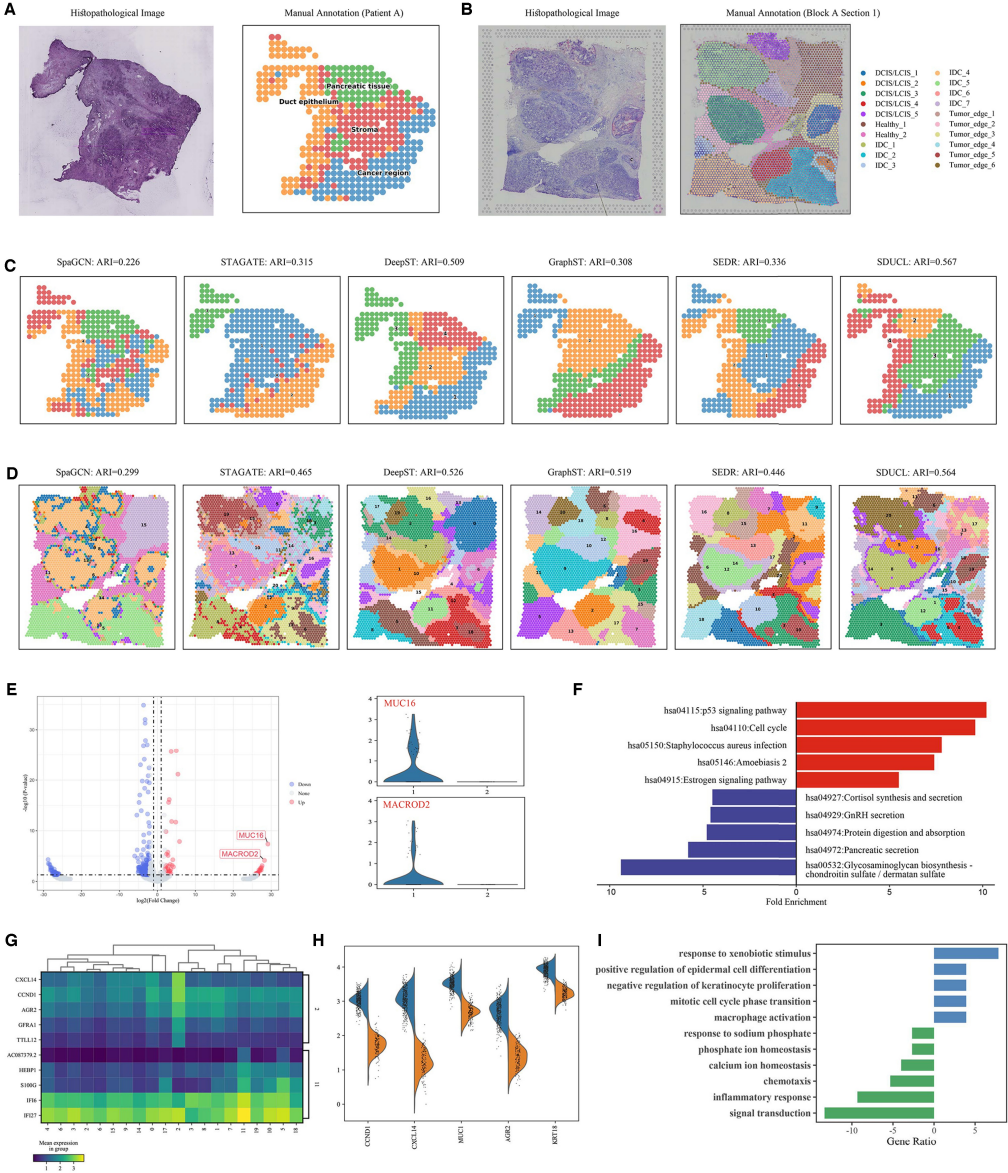
SDUCL for dissecting heterogeneity in different tumors. (A) Histological images and manual annotations of the PDAC dataset. (B) Histological images and manual annotations of the BRCA dataset. (C) Clustering results on PDAC dataset. (D) Clustering results on BRCA dataset. (E) Volcano plots illustrating differentially expressed genes between cluster 1 and cluster 2 in the PDAC dataset (left); expression patterns of MUC16 and MACROD2 between cluster 1 and cluster 2 (right). (F) Pathway enrichment analysis of DEGs between cluster 1 and cluster 2 in the PDAC dataset, where pathways upregulated in cluster 1 are indicated at the top of the image, and the opposite at bottom. (G) Heatmaps displaying gene expression profiles of the top 5 DEGs between cluster 3 and cluster 12 in the BRCA dataset. (H) Expression patterns of the top 5 differentially expressed genes between cluster 3 and cluster 12 in the BRCA dataset. (I) DEGs between cluster 3 and cluster 12 in the BRCA dataset.

PDAC is one of the more malignant tumors worldwide, characterized by its intricate tumor microenvironment (Halbrook *et al*. 2023). Moncada *et al.* (2020) initially divided the histological images into four main regions including the cancer region, non-malignant ductal epithelial region, stromal region, and pancreatic tissue region (Fig. 5A). Spatial clustering was first performed using SpaGCN, STAGATE, DeepST, GraphST, SEDR, and SDUCL. Notably, the SpaGCN did not utilize histological images due to compatibility issues with the PDAC dataset. As shown in Fig. 5C, the clustering results of DeepST and SDUCL are more consistent with the manual annotations overall, with SDUCL demonstrating superior capability in distinguishing ductal epithelium from tumor areas. Among the baseline methods, SpaGCN displayed high fragmentation; STAGATE and GraphST effectively outlined tumor areas but erroneously merged with other regions; DeepST and SEDR effectively separated pancreatic tissue from cancerous areas but inaccurately delineated boundaries between ductal epithelium and stromal regions. In terms of quantitative metrics, SDUCL performed the best, with an ARI of 0.567. Following was DeepST with an ARI of 0.509, while SEDR ranked third with an ARI of 0.336, 23.1% than SDUCL. Overall, methods considering histological images (DeepST, SDUCL) showed significantly superior performance compared to other methods on the PDAC dataset.

To explore intratumoral transcriptional differences, we performed differential gene analysis and pathway enrichment analysis between Cluster 1 (cancer region) and Cluster 2 (pancreatic tissue). We detected 434 significantly differentially expressed genes (|log fold change| ≥ 2; *P*-value < 0.05) between these clusters (Fig. 5E). In particular, the MUC16 gene has been shown to exert oncogenic effects in pancreatic ductal adenocarcinoma, with its aberrant expression enhancing the invasive phenotype of PDAC (Thomas *et al*. 2021). Subsequent pathway enrichment analysis revealed (Fig. 5F) that the upregulated pathways primarily involve the p53 signaling pathway and cell cycle, both correlated with the invasiveness of pancreatic cancer tumors (Sherr and McCormick 2002). Conversely, the downregulated pathways were primarily related to pancreatic secretion and protein digestion and absorption, indicating that the down-regulation of the pancreatic secretion pathway might affect the pancreatic endocrine function, thereby impacting normal pancreatic secretion and subsequently regulating food digestion and absorption by the pancreas. In short, SDUCL can decipher intra-tumoral heterogeneity and reveal key signaling pathways involved in tumor initiation and progression.

#### 3.2.2 Human breast cancer

Breast cancer is highly heterogeneous and encompasses a complex microenvironment, which cannot be adequately characterized solely based on manual annotations of tumor morphology alone. Therefore, Fu *et al.* manually segmented the histological images into four main morphological types and 20 subregions based on pathological features, including ductal carcinoma *in situ*/lobular carcinoma *in situ* (DCIS/LCIS), healthy tissue (Healthy), invasive ductal carcinoma (IDC), and tumor edge with low-malignancy characteristics (Fig. 5B). We applied the same baseline methods for spatial clustering as in the PDAC dataset. As shown in Fig. 5C, the six methods generally in line with manual annotation at a macroscopic level. Among them, STAGATE shows the highest degree of fragmentation, followed by SpaGCN, while the results of the remaining four methods appeared smoother and show less fragmentation. Specifically, SDUCL could clearly distinguish the IDC_3/4/5, DCIS/LCIS_1/3/4 areas. Except for SDUCL, the other methods failed to depict the IDC_4 and IDC_5 separately. In terms of quantitative comparison, SDUCL achieved the highest ARI value of 0.564.

To explore the heterogeneity of tumor tissues, we compared the top 5 differentially expressed genes between cluster 3 (representing IDC) and cluster 12 (representing DCIS/LCIS). The results indicated that there were significant expression differences among these different clusters (Fig. 5G), suggesting considerable heterogeneity among different tumor tissues. To delve deeper into the heterogeneity among tumor tissues, we further performed differential expression analysis and Gene Ontology (GO) enrichment analysis. We detected 144 significantly differentially expressed genes between Cluster 3 and Cluster 12 (|log fold change|≥2; *P*-value < 0.05). Among the top 5 differentially expressed genes (Fig. 5H), CCND1 plays a crucial role in cell proliferation and has been implicated in tumor invasion and metastasis (Fusté *et al*. 2016); the expression of CXCL14 is an independent marker of poor prognosis in breast cancer (Sjöberg *et al*. 2016); the high expression of KRT18 has been associated with deep tumor infiltration and correlates with tumor invasiveness and drug resistance, holding potential value in the diagnosis and prognostic assessment of breast cancer (Zhang *et al*. 2019). Additionally, we performed GO enrichment analysis of the differentially expressed genes (Fig. 5I) to reveal the biological processes (BPs) in which these genes were involved. The results showed that upregulated BPs were mainly involved in immune regulation, cell proliferation and macrophage activation, with Cluster 3 showing stronger association with tumor progression, invasion and metastasis (Kuroda *et al*. 2021); downregulated BPs were associated with immune response, with Cluster 12 showing a weakened response to inflammatory stimuli, which may lead to impaired immune function or inhibition of the inflammatory process, revealing results consistent with Xu *et al.* (2024). These results suggest differences in function and regulation among different tumor tissues. Therefore, SDUCL aids in dissecting the heterogeneity among tumors and enriches our understanding of spatial transcriptomic data with a more unique perspective.

### 3.3 Ablation study

To further validate the effectiveness of each key component of SDUCL, a series of ablation experiments were conducted. In these experiments, we removed the image feature extraction module (w/o img), spatial feature extraction module (w/o katz), tensor fusion module (w/o fusion), signal diffusion microenvironment discovery module (w/o signal), as well as the reconstruction loss (w/o rec) and contrastive loss (w/o con). The DLPFC dataset was used with the same evaluation metrics as above (ARI, NMI, V-Measure, Purity) to observe the specific impact of the different modules on experiment performance.

**Figure 6.**
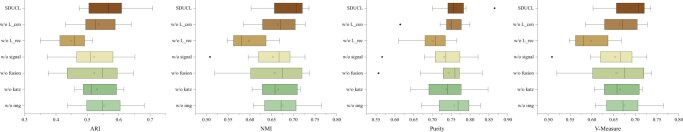
Boxplots of SDUCL ablation experiment results on the DLPFC dataset.

Firstly, the removal of the image feature extraction module led to a 1.2% decrease in the mean ARI and a 1.8% decrease in the median ARI. Secondly, when the spatial feature extraction module was removed, the average and median ARI decreased by 3.5% and 5.5%, respectively. Thirdly, the mean and median of ARI decrease by 4.4% and 1.8% when the tensor fusion module was removed. After removing the signal diffusion microenvironment discovery module, the mean and median ARI dropped by 4.6% and 5.6%, respectively. Additionally, removing the reconstruction loss led to a significant decrease of 10.2% and 10.9% in the mean and median ARI, while the removal of contrastive loss caused a 3% decrease in both metrics. Similar trends were observed across other evaluation metrics (Fig. 6). Overall, the ablation experiments highlight the importance of each component, particularly the reconstruction loss and the signal diffusion microenvironment discovery module. SDUCL effectively utilizes image information as well as spatial topology information, and fully integrates spatial and feature information to ultimately enhance model performance.

## 4 Discussion and conclusion

In this study, we propose SDUCL, an unsupervised contrastive learning framework based on signal diffusion. SDUCL comprehensively considers image features, spatial topology features, and gene expression information. It integrates these extracted features into a tensor to obtain an augmented feature representation, and finally learns a low-dimensional representation of each cell/spot by an unsupervised contrastive learning strategy based on signal diffusion. Existing contrastive learning methods typically aggregate only directly adjacent nodes as global representations, neglecting cell interactions within the microenvironment. To overcome this limitation, we developed a signal diffusion microenvironment discovery algorithm to identify the microenvironments of cells/spots in the spatial relationship graph. SDUCL aggregates the representations of nodes within these microenvironments to form a global node representation. By maximizing the mutual information between the local and global representations of nodes, SDUCL is able to learn discriminative low-dimensional embeddings.

We have evaluated the effectiveness of SDUCL on both normal and tumor datasets, and its learned low-dimensional representations performed well in a variety of downstream tasks such as clustering, visualization, trajectory inference, and differential gene analysis. However, SDUCL currently incurs high memory consumption, which limits its application to large-scale spatial transcriptomic datasets. In the future, we plan to optimize the algorithm performance using mini-batch processing and parallel computing. In addition, we aim to extend the downstream tasks of SDUCL to enhance its functionality and broaden its application scenarios.

## Supplementary Material

btae663_Supplementary_Data

## Data Availability

All datasets used in this article are publicly released and available for download. (1) Human dorsolateral prefrontal cortex dataset (http://spatial.libd.org/spatialLIBD). (2) Mouse brain serial section (https://support.10xgenomics.com/spatial-gene-expression/datasets). (3) Phalaenopsis dataset (https://db.cngb.org/stomics/datasets/STDS0000149). (4) Dataset and annotation for human breast cancer (https://support.10xgenomics.com/spatial-gene-expression/datasets/1.1.0/V1_Breast_Cancer_Block_A_Section_1 and https://github.com/JinmiaoChenLab/SEDR). (5) Dataset and Annotation for Human Pancreatic Ductal Adenocarcinoma (http://cancersrt.info/Download and https://www.ncbi.nlm.nih.gov/geo/query/acc.cgi?acc=GSE111672). (6) Mouse olfactory bulb tissue dataset (https://github.com/JinmiaoChenLab/SEDR). (7) Mouse hypothalamic preoptic dataset (http://sdmbench.drai.cn/).

## References

[btae663-B1] Allen WE , Blosser TR, Sullivan ZA et al Molecular and spatial signatures of mouse brain aging at single-cell resolution. Cell 2023;186:194–208.e18. 36580914 10.1016/j.cell.2022.12.010 PMC10024607

[btae663-B2] Cai J , Liu X, Vanneste K et al The genome sequence of the orchid phalaenopsis equestris. Nat Genet 2015;47:65–72. 25420146 10.1038/ng.3149

[btae663-B3] Chen A , Liao S, Cheng M et al Spatiotemporal transcriptomic atlas of mouse organogenesis using DNA nanoball-patterned arrays. Cell 2022;185:1777–92.e21. 35512705 10.1016/j.cell.2022.04.003

[btae663-B4] Chen KH , Boettiger AN, Moffitt JR et al Spatially resolved, highly multiplexed RNA profiling in single cells. Science 2015;348:aaa6090. 25858977 10.1126/science.aaa6090 PMC4662681

[btae663-B5] Dong K , Zhang S. Deciphering spatial domains from spatially resolved transcriptomics with an adaptive graph attention auto-encoder. Nat Commun 2022;13:1739. 35365632 10.1038/s41467-022-29439-6 PMC8976049

[btae663-B6] Dries R , Zhu Q, Dong R et al Giotto: a toolbox for integrative analysis and visualization of spatial expression data. Genome Biol 2021;22:78. 33685491 10.1186/s13059-021-02286-2 PMC7938609

[btae663-B7] Fusté NP , Fernández-Hernández R, Cemeli T et al Cytoplasmic cyclin d1 regulates cell invasion and metastasis through the phosphorylation of paxillin. Nat Commun 2016;7:11581. 27181366 10.1038/ncomms11581 PMC4873647

[btae663-B8] Halbrook CJ , Lyssiotis CA, Pasca di Magliano M et al Pancreatic cancer: advances and challenges. Cell 2023;186:1729–54. 37059070 10.1016/j.cell.2023.02.014 PMC10182830

[btae663-B9] He K, Zhang X, Ren S et al Deep residual learning for image recognition. In: *Proceedings of the IEEE Conference on Computer Vision and Pattern Recognition*. 2016, 770–8.

[btae663-B10] Hsu H-F , Chen W-H, Shen Y-H et al Multifunctional evolution of b and agl6 mads box genes in orchids. Nat Commun 2021;12:902. 33568671 10.1038/s41467-021-21229-w PMC7876132

[btae663-B11] Hu J , Li X, Coleman K et al SpaGCN: integrating gene expression, spatial location and histology to identify spatial domains and spatially variable genes by graph convolutional network. Nat Methods 2021;18:1342–51. 34711970 10.1038/s41592-021-01255-8

[btae663-B12] Kuroda H , Jamiyan T, Yamaguchi R et al Tumor microenvironment in triple-negative breast cancer: the correlation of tumor-associated macrophages and tumor-infiltrating lymphocytes. Clin Transl Oncol 2021;23:2513–25. 34089486 10.1007/s12094-021-02652-3 PMC8557183

[btae663-B13] Li J , Chen S, Pan X et al Cell clustering for spatial transcriptomics data with graph neural networks. Nat Comput Sci 2022;2:399–408. 38177586 10.1038/s43588-022-00266-5

[btae663-B14] Liu C , Leng J, Li Y et al A spatiotemporal atlas of organogenesis in the development of orchid flowers. Nucleic Acids Res 2022;50:9724–37. 36095130 10.1093/nar/gkac773 PMC9508851

[btae663-B15] Long Y , Ang KS, Li M et al Spatially informed clustering, integration, and deconvolution of spatial transcriptomics with graphst. Nat Commun 2023;14:1155. 36859400 10.1038/s41467-023-36796-3 PMC9977836

[btae663-B16] Martínez V , Berzal F, Cubero J-C et al A survey of link prediction in complex networks. ACM Comput Surv 2017;49:1–33.

[btae663-B17] Maynard KR , Collado-Torres L, Weber LM et al Transcriptome-scale spatial gene expression in the human dorsolateral prefrontal cortex. Nat Neurosci 2021;24:425–36. 33558695 10.1038/s41593-020-00787-0 PMC8095368

[btae663-B18] Moncada R , Barkley D, Wagner F et al Integrating microarray-based spatial transcriptomics and single-cell RNA-seq reveals tissue architecture in pancreatic ductal adenocarcinomas. Nat Biotechnol 2020;38:333–42. 31932730 10.1038/s41587-019-0392-8

[btae663-B19] Moses L , Pachter L. Museum of spatial transcriptomics. Nat Methods 2022;19:534–46. 35273392 10.1038/s41592-022-01409-2

[btae663-B20] Pham D, Tan X, Balderson B et al Robust mapping of spatiotemporal trajectories and cell–cell interactions in healthy and diseased tissues. Nature communications 2023;14:7739. 10.1038/s41467-023-43120-6 PMC10676408 38007580

[btae663-B21] Polyak K. Heterogeneity in breast cancer. J Clin Invest 2011;121:3786–8. 21965334 10.1172/JCI60534 PMC3195489

[btae663-B22] Rao A , Barkley D, França GS et al Exploring tissue architecture using spatial transcriptomics. Nature 2021;596:211–20. 34381231 10.1038/s41586-021-03634-9 PMC8475179

[btae663-B23] Renelt M , von Bohlen und Halbach V, von Bohlen und Halbach O et al Distribution of pcp4 protein in the forebrain of adult mice. Acta Histochem 2014;116:1056–61. 24954028 10.1016/j.acthis.2014.04.012

[btae663-B24] Satija R , Farrell JA, Gennert D et al Spatial reconstruction of single-cell gene expression data. Nat Biotechnol 2015;33:495–502. 25867923 10.1038/nbt.3192 PMC4430369

[btae663-B26] Sherr CJ , McCormick F. The Rb and p53 pathways in cancer. Cancer Cell 2002;2:103–12. 12204530 10.1016/s1535-6108(02)00102-2

[btae663-B27] Sjöberg E , Augsten M, Bergh J et al Expression of the chemokine cxcl14 in the tumour stroma is an independent marker of survival in breast cancer. Br J Cancer 2016;114:1117–24. 27115465 10.1038/bjc.2016.104 PMC4865967

[btae663-B28] Tan X , Su A, Tran M et al Spacell: integrating tissue morphology and spatial gene expression to predict disease cells. Bioinformatics 2020;36:2293–4. 31808789 10.1093/bioinformatics/btz914

[btae663-B29] Thomas D , Sagar S, Liu X et al Isoforms of muc16 activate oncogenic signaling through EGF receptors to enhance the progression of pancreatic cancer. Mol Ther 2021;29:1557–71. 33359791 10.1016/j.ymthe.2020.12.029 PMC8058431

[btae663-B30] Velickovic P et al Deep graph infomax. ICLR (Poster) 2019;2:4.

[btae663-B32] Xu C , Jin X, Wei S et al Deepst: identifying spatial domains in spatial transcriptomics by deep learning. Nucleic Acids Res 2022;50:e131. 36250636 10.1093/nar/gkac901 PMC9825193

[btae663-B33] Xu H , Fu H, Long Y et al Unsupervised spatially embedded deep representation of spatial transcriptomics. Genome Med 2024;16:12. 38217035 10.1186/s13073-024-01283-x PMC10790257

[btae663-B34] Yang Y , Shi X, Liu W et al Sc-meb: spatial clustering with hidden Markov random field using empirical bayes. Brief Bioinform 2022;23:bbab466. 34849574 10.1093/bib/bbab466 PMC8690176

[btae663-B35] Yu X , Li W, Wang J et al Construction of gene expression patterns to identify critical genes under SARS-CoV-2 infection conditions. IEEE/ACM Trans Comput Biol Bioinform2024a;21:607–18. 37285245 10.1109/TCBB.2023.3283534

[btae663-B36] Yu X , Li W, Yang B et al Periodic distribution entropy: unveiling the complexity of physiological time series through multidimensional dynamics. Inf Fusion2024b;108:102391.

[btae663-B37] Zeng Y , Wei Z, Yu W et al Spatial transcriptomics prediction from histology jointly through transformer and graph neural networks. Brief Bioinform 2022;23:bbac297. 35849101 10.1093/bib/bbac297

[btae663-B38] Zeng Y , Yin R, Luo M et al Identifying spatial domain by adapting transcriptomics with histology through contrastive learning. Brief Bioinform 2023;24:bbad048. 36781228 10.1093/bib/bbad048

[btae663-B39] Zhang J , Hu S, Li Y et al Krt18 is correlated with the malignant status and acts as an oncogene in colorectal cancer. Biosci Rep 2019;39:BSR20190884. 31345960 10.1042/BSR20190884 PMC6692566

[btae663-B40] Zhao E , Stone MR, Ren X et al Spatial transcriptomics at subspot resolution with Bayes space. Nat Biotechnol 2021;39:1375–84. 34083791 10.1038/s41587-021-00935-2 PMC8763026

[btae663-B41] Zong Y, Yu T, Wang X et al const: an interpretable multi-modal contrastive learning framework for spatial transcriptomics. bioRxiv, 10.1101/2022.01.14.476408, 2022, preprint: not peer reviewed.

