# Supplementary material for: A signal-diffusion-based unsupervised contrastive representation learning for spatial transcriptomics analysis
Source: Bioinformatics. 2024 Nov 15;40(11):btae663. doi: 10.1093/bioinformatics/btae663 (PMC11588211; doi:10.1093/bioinformatics/btae663)
Supplement: btae663_Supplementary_Data [file btae663_supplementary_data.docx]

**Supplementary Material**

**Supplementary Methods**

SA1 Data preprocessing

For all datasets, a series of preprocessing steps were performed on the raw gene expression matrices using the Scanpy package. First, a logarithmic transformation (Eq. (1)) was performed to transform the data into logarithmic space. This helps to reduce data bias and compress extreme values. Next, normalization (Eq. (2)) is performed for each gene to ensure that the expression level of each gene has zero mean and unit variance, which eliminates scale differences between different genes. Finally, to improve the robustness of the model, we selected the top 3000 highly variable genes as model input.

$X=\log\left( x+1 \right)$ （1）

$Z=\frac{X-E(X)}{\sigma\left( X \right)}$ （2）

where x is the original gene expression matrix, X is the log-transformed gene expression, and $E(X)$ and $\sigma\left( X \right)$ denote the mean and standard deviation of the gene expression matrix.

SA2 Baseline and competing methods

***Seurat.*** Seurat, a widely employed toolkit in single-cell RNA sequencing studies, has undergone enhancements in its SeuratV5 version to cater to spatial transcriptomic analyses. However, it is worth noting that this approach does not fully leverage spatial information.

***Giotto.*** Giotto connects cells that are physically close by constructing spatial grids and neighborhood networks. It utilizes a hidden Markov random field (HMRF) model to explore spatial dependencies and identify spatial domains with consistent gene expression patterns.

***BayesSpace.*** BayesSpace employs a fully Bayesian statistical approach, utilizing multivariate t-distributions to model low-dimensional representations of gene expression, capturing latent structures and variability within the data. Subsequently, spatial information is integrated through a Potts model, enhancing clustering analysis by leveraging information from spatial neighborhoods.

***stLearn.*** stLearn introduces the Spatial Gene Expression Mean-normalization (SME) strategy for spatial gene expression normalization. This strategy integrates neighborhood information and morphological distances to normalize gene expression data, enabling unsupervised clustering analysis.

***SpaGCN.*** SpaGCN integrates histological, spatial positional, and gene expression data to identify spatial domains. It constructs spatially weighted graphs of cells by combining spatial coordinates and morphological features, and then applies graph convolutional networks to merge this spatially weighted graph with gene expression data for clustering.

***DeepST.*** DeepST leverages pre-trained deep neural network model ResNet to extract image features from histopathological information (H&E stained images), integrating image features, gene expression, and spatial location data to generate a spatially enhanced gene expression matrix. It adopts a joint generation approach utilizing graph neural network encoders and denoising autoencoders to generate latent representations of enhanced ST data.

***STAGATE.*** STAGATE integrates spatial information and gene expression to learn low-dimensional latent embeddings. It adaptively learns the similarity between neighboring points by incorporating an attention mechanism, while also denoising the data while preserving spatial expression patterns.

***GraphST.*** GraphST combines graph neural networks with self-supervised contrastive learning. It employs graph convolutional networks as encoders to iteratively integrate gene expression information from neighboring points. Through self-supervised contrastive learning on graphs, it minimizes the embedding distance between spatially adjacent points.

***SEDR.*** SEDR employs a joint training approach utilizing masked autoencoders and variational autoencoders to learn the latent low-dimensional representation of gene expression embedding spatial information. The masked autoencoder is utilized for learning gene representations, while the variational autoencoder is employed for embedding spatial information.

SA3 Clustering

For the low-dimensional representations obtained by SDUCL, various clustering methods are supported to cluster them. In this study, we use mclust or kmeans for clustering spatial transcriptome data with a priori information, and we use the Leiden or Louvain algorithms for data without a priori information. It is worth noting that when applying the Leiden or Louvain algorithms, we try different resolutions to explore the best clustering results.

**SA4 Hyper-parameters setting**

The framework of this study was implemented via Pytorch. The image feature extractor utilized ResNet50 from torchvision.models with default pre-training weights. The encoder hidden layer dimensions were set to [512,64], with 1000 training epochs, and an Adam optimizer with a learning rate of 0.001.

The experiments were run on a server equipped with an Intel(R) Xeon(R) Silver 4210 CPU @ 2.20GHz processor, 64GB DDR4 RAM, and NVIDIA GeForce RTX 4090 GPU. The operating system used was Ubuntu 20.04 LTS. All experimental code was executed within a Python 3.10 environment.

**SA5 Evaluation metrics for clustering**

For spatial transcriptomic data with manual annotations, we will evaluate the spatial clustering performance using four commonly used clustering metrics, including the Adjusted Rand Index (ARI), Normalized Mutual Information (NMI), V-measure, and Purity.

ARI measures the consistency between clustering results and true labels by evaluating whether pairs of samples are correctly assigned to the same or different clusters.

$ARI=\frac{\sum_{ij} ({n_{ij} \atop2})-[\sum_{i} ({a_{i} \atop2})\sum_{j} ({b_{j} \atop2})]/({n \atop2})}{\frac{1}{2}[\sum_{i} ({a_{i} \atop2})+\sum_{j} ({b_{j} \atop2})]-[\sum_{i} ({a_{i} \atop2})\sum_{j} ({b_{j} \atop2})]/({n \atop2})}$ (3)

NMI evaluates the mutual information between clustering results and true labels, quantifying the dependency between the two.

$NMI=\frac{-2\sum_{ij} n_{ij}log(\frac{n_{ij}n}{a_{i}b_{j}})}{\sum_{i} a_{i}\log\frac{a_{i}}{n}+\sum_{j} b_{j}\log\frac{b_{j}}{n}}$ (4)

V-measure is the harmonic mean of two complementary clustering properties: homogeneity (each cluster contains only samples from one class) and completeness (all samples of the same type are grouped in the same cluster)

$V-measure=\frac{2\times(homogeneity\times completeness)}{(homogeneity+completeness)}$ (5)

Purity measures the extent to which each cluster contains samples from primarily one class.

$Purity=\frac{1}{n}\sum_{i} \max_{j}\left| C_{i}\cap L_{j} \right|$ (6)

where $C_{i}$ represents the $i-th$ cluster, $L_{j}$denotes the $j-th$ true class,$n_{ij}$indicates the number of samples simultaneously belonging to cluster$c_{i}$ and class $L_{j}$, $a_{i}$represents the number of samples in cluster$c_{i}$, $b_{j}$signifies the number of samples in class$L_{j}$, $n$ denotes the total number of samples. For each of these metrics, a higher value indicates better clustering performance.

In cases lacking manual annotations, we employ two internal evaluation metrics, namely the Silhouette Coefficient (SC) and Davies-Bouldin Index (DBI), to assess clustering.

$SC=\frac{1}{n}\sum_{i=1}^{n} \frac{b_{i}-a_{i}}{max\{a_{i},b_{i}\}}$ (7)

where $a_{i}$ denotes the average distance from sample $i$to others within the same cluster, and$b_{i}$represents the average distance from sample$i$ to samples in the nearest other cluster. The value of SC closer to 1 signifies compact and well-separated clusters.

$DBI=\frac{1}{k}\sum_{i=1}^{k} \max_{j\neq i}\left( \frac{\sigma_{i}+\sigma_{j}}{d\left( c_{i},c_{j} \right)} \right)$ (8)

where $k$ represents the number of clusters, $c_{i}$ denotes the centroid of the$i-th$ cluster, $\sigma_{i}$represents the average distance from samples in cluster$i$to its centroid, and$d\left( c_{i},c_{j} \right)$ indicates the distance between cluster centroids. A lower DBI value indicates better clustering performance.

**SA6 Analysis of shuffle robustness with different random seed**

To generate negative samples, we created corrupted graphs by randomly shuffling the feature vectors of the nodes while preserving the original graph's topology. To evaluate the potential impact of random shuffling on model performance, we conducted experiments on 12 slices from the DLPFC dataset, using 10 different random seeds (ranging from 42 to 16,384) to introduce randomness. Each random seed was used to independently shuffle the data, followed by model training, with the performance on each slice recorded to assess its stability.

The experimental results, as shown in Supplementary Figure S1, the ARI scores for each data slice were recorded under different random seed conditions. The boxplot illustrates that despite the introduction of randomness, SDUCL’s performance exhibited minimal fluctuations, indicating that random shuffling had no significant impact on model performance, and it maintained stability across different random conditions.

**SA7 Impact of Shuffling Ratio on Model Performance**

To evaluate the model's performance under different shuffling ratios, we progressively adjusted the degree of node feature shuffling during the generation of negative samples, ranging from partial shuffling (ratio of 0.1) to complete shuffling (ratio of 1.0), and observed the resulting performance changes.

The experimental results show that at lower shuffling ratios (0.1 to 0.7), there was still some overlap between the negative and positive samples, which made it more difficult for the model to distinguish between them, leading to a drop in performance. This indicates that the feature differences between positive and negative samples were not yet pronounced enough to fully separate them. However, as the shuffling ratio increased to 0.8 and above, model performance improved significantly, reaching its peak at a ratio of 1.0 (Supplementary Figure S2). This suggests that when the node features are fully randomized, the distinction between positive and negative samples is maximized, allowing the model to perform contrastive learning more effectively and better capture the distinguishing features between the two.

Overall, increasing the shuffling ratio gradually amplifies the differences between positive and negative samples, with the model achieving optimal performance at a complete shuffle (ratio of 1.0). This demonstrates that fully shuffling the node features to generate negative samples can significantly enhance the model's ability to perform contrastive learning, thereby improving its ability to distinguish between positive and negative samples and enhancing its overall performance.

**SA8 Hyperparameter analysis**

To evaluate the impact of different hyperparameters on model performance, we conducted a hyperparameter analysis based on the DLPFC dataset. The hyperparameters were divided into two categories: the first category is related to the loss function, including the self-reconstruction loss weight $\alpha$ (range: 1 to 50, with a step size of 5) and the contrastive loss weight β (range: 1 to 5, with a step size of 0.5); the second category is related to the training process, including the learning rate $lr$ (range: $5\times{10}^{-5}$ to 1) and the number of training epochs (range: 100 to 2000, with a step size of 250). In each experiment, we systematically varied one hyperparameter at a time while holding the others constant to assess its independent influence on model performance.

The experimental results indicate that as the number of training epochs increases, model performance improves progressively, reaching an optimal point around 1000 epochs. Beyond this point, further increases in training epochs may lead to overfitting (**Figure S7A**). The model achieves its best performance when the learning rate is set to 0.001, with both higher and lower learning rates resulting in performance degradation (**Figure S7B**). An analysis of the hyperparameters $\alpha$ and $\beta$ reveals that model performance remains stable and robust when $\alpha$ is within the range [1, 15] and $\beta$ is between [1, 3]. This suggests that the model is resilient to variations in hyperparameters within these ranges. The model achieves optimal performance specifically when $\alpha=5$ and $\beta=1.5$ (**Figure S7C**).

**Supplementary Figures**


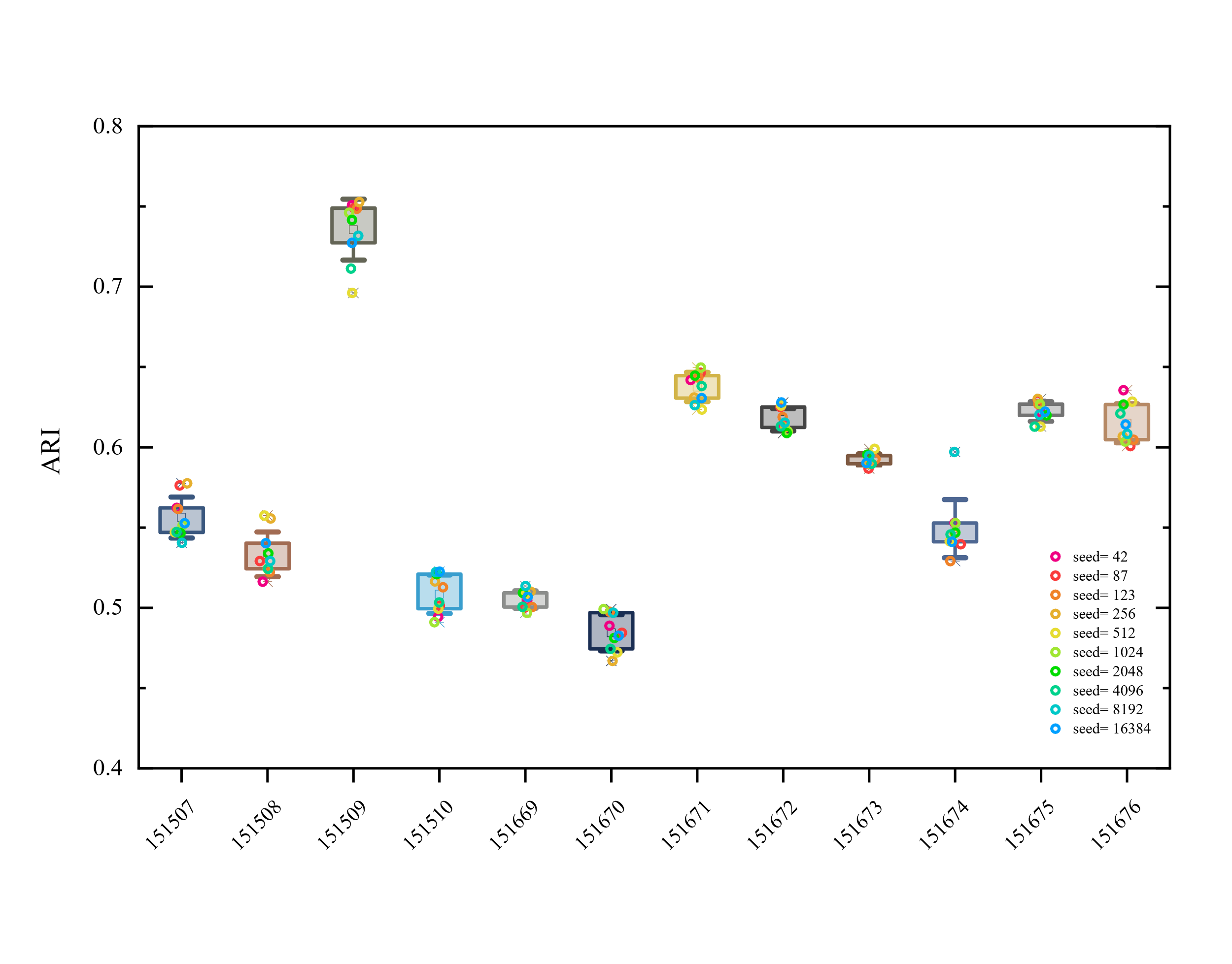


**Supplementary Figure S1.** ARI scores across different random seeds for 12 slices from the DLPFC dataset


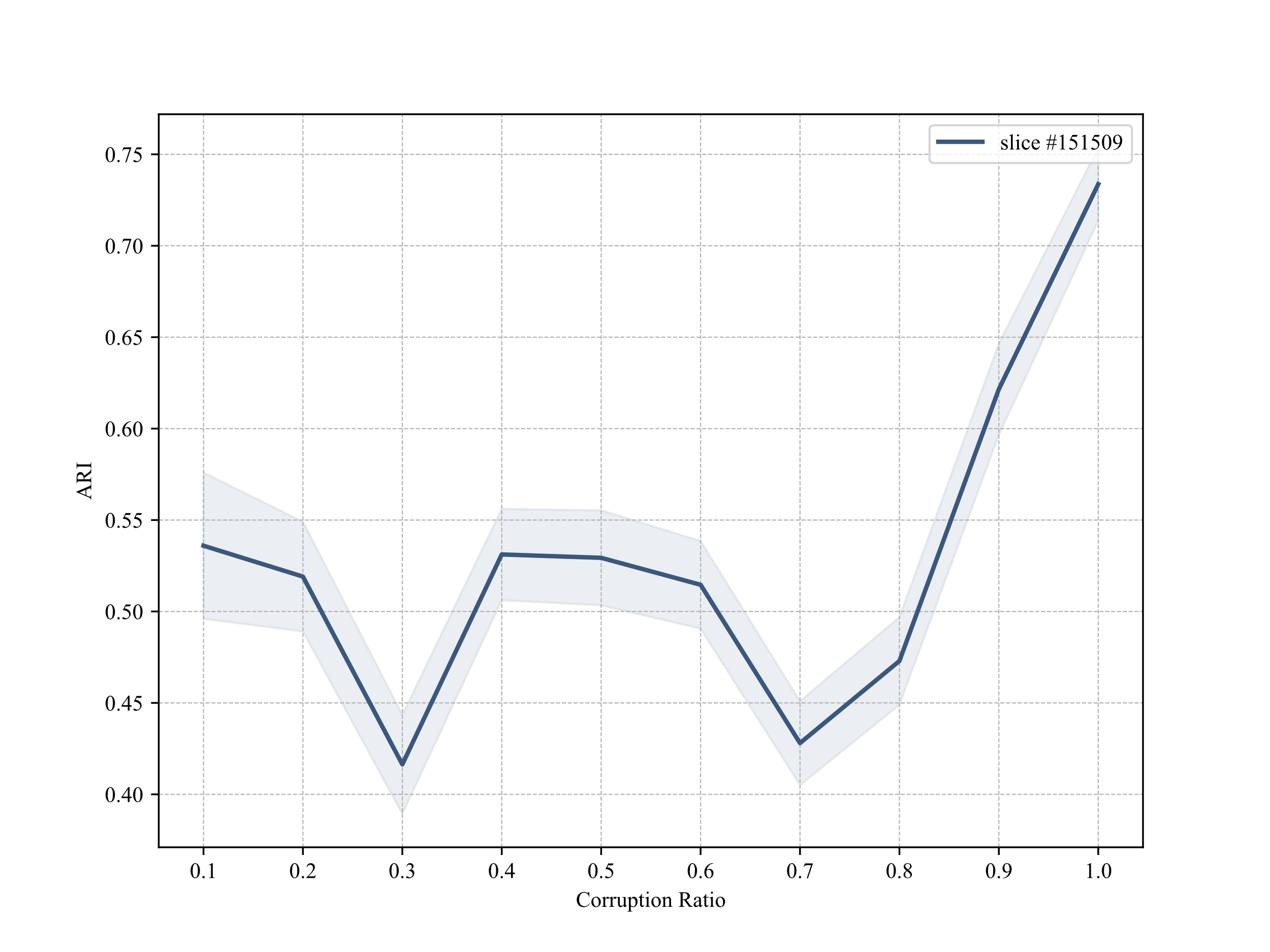


**Supplementary Figure S2.** ARI scores across different corruption ratio for slice #151509 from the DLPFC dataset

**Supplementary Figure S3.** Boxplots of clustering evaluation metrics for 12 slices from the DLPFC, showcasing the ARI, NMI, purity and V-measure

**Supplementary Figure. S4.** Spatial clustering results of slice 1 from phalaenopsis dataset using SpaGCN, DeepST, STAGATE, GraphST, SEDR and SDUCL

**Supplementary Figure S5.** Spatial clustering results of slice 2 from phalaenopsis dataset using SpaGCN, DeepST, STAGATE, GraphST, SEDR and SDUCL


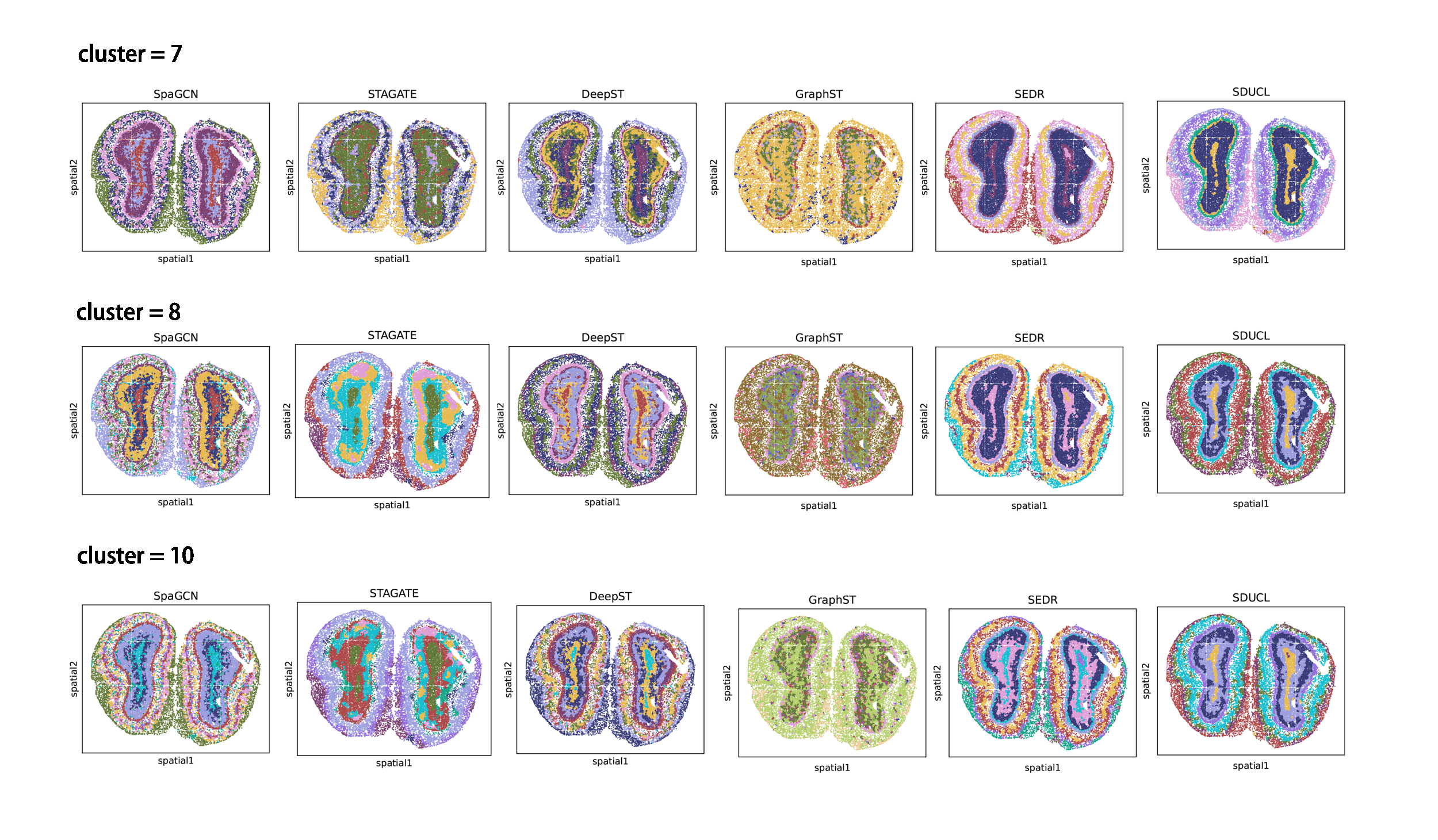
 **Supplementary Figure S6** Clusters identified in the mouse olfactory bulb Stereo-seq dataset using six methods (SpaGCN, STAGATE, DeepST, GraphST, SEDR, and SDUCL)—with the number of clusters set to 7, 8, and 10, respectively.

**Supplementary Figure S7** Hyperparameter analysis for model performance on the DLPFC Dataset. (A) Effect of training epochs on SDUCL (B) Effect of learning rate on SDUCL (C) Effect of the loss function hyperparameters $\alpha$and $\beta$ on SDUCL
